# Editorial: Precision Medicine: Impact of Cytochromes P450 and Transporters Genetic Polymorphisms, Drug-Drug Interactions, Disease on Safety and Efficacy of Drugs

**DOI:** 10.3389/fphar.2021.834717

**Published:** 2022-01-18

**Authors:** Youssef Daali, Amin Rostami-Hodjegan, Caroline F. Samer

**Affiliations:** ^1^ Division of Clinical Pharmacology and Toxicology, Department of Anesthesiology, Pharmacology, Intensive Care and Emergency Medicine, Geneva University Hospitals, Geneva, Switzerland; ^2^ Institute of Pharmaceutical Sciences of Western Switzerland (ISPSO), University of Geneva, Geneva, Switzerland; ^3^ Laboratory of Clinical Pharmacology, Faculty of Medicine, University of Geneva, Geneva, Switzerland; ^4^ Centre for Applied Pharmacokinetic Research, School of Health Sciences, University of Manchester, Manchester, United Kingdom; ^5^ Certara, Princeton, NJ, United States

**Keywords:** pharmacokinetics, personalized & precision medicine (PPM), pharmacogenenomics and personalised medicine, drug-drug interactions, PBPK

The idealistic concept “one-size fits all” in drug treatment has faced the challenge of variable patient response over several decades. The concept has gradually been replaced by precision dosing, under the umbrella of precision medicine, where patients receive individually tailored therapy to minimize the risk of potential adverse drug effects or inefficacy (Darwich et al., 2021). A large interindividual variability in response to treatment is frequently observed due to the variety of genetic and environmental factors. The covariates determining the variable pharmacodynamics are not adequately studied. However, the individual attributes defining pharmacokinetic variability are well established. These play an important role in precision dosing and involve factors such as genetic polymorphisms of phase I and phase II drug metabolizing enzymes, drug-drug interactions (DDI), the modulating effect of diseases itself on function, and activity of enzymes and transporters.

Identification of the main factors influencing the activity of enzymes and transporters at the individual level is essential for tailored therapy. However, the complex interaction between multiple factors leading to complex drug-drug-gene-disease interactions is difficult to predict, with occasionally fatal consequences (Storelli et al., 2018). Therefore, there is an urgent need to increase applications of the knowledge gathered so far regarding these sources of variability into clinical practice. The use of model informed precision dosing (MIPD) alongside better patient characterization are powerful tools that help clinicians in individualized patient care. These computer-based modeling and simulation techniques can integrate information on individual capacity for enzymes and transporters alongside many other factors to predict a drug dose for a given patient and manage complex drug-drug-gene-disease scenarios (Polasek et al., 2019). Hence, the current issue of the journal is devoted to the Research Topic of precision dosing and the impact of cytochromes P450 (CYPs), transporters genetic polymorphisms, drug-drug interactions, and disease, on the safety and efficacy of drugs.

In oncology, the development of tyrosine kinase inhibitors (TKI) has revolutionized anticancer targeted therapy by increasing patient survival rates, particularly in hematologic neoplasms. However, treatment failure is observed in 20–25% of chronic myeloid leukemia (CML) patients due to mutations in the BCR-ABL1 gene or other factors not related to the fusion gene such as CYP3A4/5 and transporters (OCT1, ABCB1, ABCG2) as reported by Kaehler and Cascorbi. The authors reviewed all pharmacogenetic aspects related to impaired TKI response in CM, discuss BCR-ABL1-dependent mechanisms as well as mutations in PK pathways (CYPs and transporters), and the role of alternative signaling pathways. The majority of these targeted drugs are metabolized by CYP3A4/5 leading to DDI with inhibitors or inducers. Molenaar-Kuijsten et al. reviewed all potential DDIs with oral targeted anticancer drugs and provided recommendations for clinical practice on how to deal with DDIs. The authors pointed out the large interindividual variability in the PK of the studied drugs with a range of 23–78%, which was reflected in the variability of the effects of CYP3A4/5 inhibitors and inducers. This variability in exposure could partly be explained by the highly variable CYP3A4/5 activity, which is 60–90% genetically determined (Ozdemir et al., 2000). Moreover, the importance of the metabolic pathway and the presence of active metabolites should be considered in the interpretation and recommendation in the clinical setting. Therefore, more complex drug-drug-genetic (DDGI) interactions should be considered.

Yang et al. also discuss the DDGI between tacrolimus and nifedipine, CYP3A5 competitive inhibitor, and the influence of CYP3A5 genotype. The co-administrated nifedipine in renal transplant patients carrying CYP3A5*3/*3 allele significantly increased tacrolimus concentrations. The authors conclude that tacrolimus personalized therapy, accounting for CYP3A5 genotype detection as well as therapeutic drug monitoring, is necessary when there is a risk of DDI. In line with these findings, Srinivas et al. evaluated the effect of CYP3A5, CYP3A4, and ABCB1 gene polymorphisms on tacrolimus trough concentrations in South Indian renal transplant patients and developed a genotype-based dosing equation to calculate the required starting daily dose of tacrolimus. The authors also investigated the effect of these genes on toxicity and organ rejections after tacrolimus administration. Transporters are also an important determinant in the variability of drugs response. They are expressed in many vital organs such as the liver and kidneys and barriers like brain-blood-barrier and intestines. Like metabolic enzymes, transporters’ activity (influx or efflux) is affected by genetic polymorphisms and DDI. El Biali et al. nicely demonstrated, using PET imaging, the influence of ABCB1 and ABCG2 and the ABCG2 c.421C > A genotype on the distribution of substrates of these transporters to the human retina. Subjects undergoing treatment with potentially retinotoxic drugs need to be carefully genotyped for this SNP and any DDI checked to avoid the potential accumulation of toxic drugs because of these complex gene-drug-drug interactions.

Villapalos-García et al. studied the effects of CYPs and transporters polymorphisms on the variability of dutasteride and tamsulosin, a combination therapy for the management of prostatic hyperplasia. Tamsulosin pharmacokinetics were very sensitive to the activity of CYP2D6 and significant associations were observed between dutasteride exposure and CYP3A4 and CYP3A5 genotypes and between tamsulosin and ABCG2, CYP3A5, and SLC22A1 genotypes. Lenoir et al. conducted a systematic review to assess another source of variation in CYP activity, inflammation. In total, 218 studies and case reports were retrieved and classified into 14 different sources of inflammation. They found that the impact of inflammation on CYP activities was isoform-specific and dependent on the nature and severity of the underlying disease causing the inflammation.

There are two approaches available for CYPs activity assessment. One involves the projection of activity based on established links either to genotyping (very common) or liquid biopsy (only started recently) (Achour et al., 2021). The other approach is a direct assessment of activity (phenotyping) using validated probes singularly or as part of a cocktail. CYPs phenotyping gives information on enzymes activity not only related to genetic variation and abundance but also on environmental and endogenous variables affecting the activity of a given level of expression for certain genotypes (Samer et al., 2013).

Ing Lorenzini et al. evaluated the concordance in CYPs activity between genotype and phenotype in the clinical setting using a Geneva micrococktail. A total of 241 patients underwent simultaneous genotyping and phenotyping and except for poor metabolizers where a perfect correlation between phenotype and genotype was observed, discrepancies were observed for the other phenotypic groups (intermediate, normal ad ultra-rapid) and not always explained by DDI. Other personal factors such as disease, inflammation, or environmental factors like food or exposition to toxins could affect CYPs activity. Interestingly, genotype and/or phenotype results explained the clinical event in 44% of cases, demonstrating the clinical utility of these tests.

MIPD using PBPK or POPPK is increasingly used in a clinical setting to help in the management of complex drug-drug-gene-disease interactions. Abouir et al. reviewed all PBPK models available for predicting DDIs and integrating intrinsic and extrinsic factors such as genetic polymorphisms and pathologies.

The editors of this Research Topic would like to thank all the participating authors for their involvement in the success of this issue and the quality of research papers and reviews submitted.
